# Fifth Annual Report State Board of Health of Penna.

**Published:** 1891-11

**Authors:** 


FIFTH ANNUAL REPORT OF THE STATE BOARD OF HEALTH AND VITAL STATISTICS OF THE COMMONWEALTH OF PENNSYLVANIA. Transmitted to the Governor December 2d, 1889. Harrisburg: Henry K. Meyers, State Printer, 1891.

This public document contains the history of the work of the State Board of Health in looking after the welfare of the people of Pennsylvania for the year 1889. It is interesting particularly in showing sources of pollution in certain outbreaks of typhoid fever and in its history of sanitary work at Johnstown after the fearful flood there in 1889. It contains also a mass of other information highly valuable to the student and statistician.

